# Supplementary material for: Chromothripsis during telomere crisis is independent of NHEJ, and consistent with a replicative origin
Source: Genome Res. 2019 May;29(5):737–49. doi: 10.1101/gr.240705.118 (PMC6499312; doi:10.1101/gr.240705.118)
Supplement: Supplemental Material [file supp_gr.240705.118_Supplemental_file_1.zip › contigs/annotated_contigs/DB51/contig.2.DB51_length_581_mean_cov_4.28743545611.docx]

**DB51_length_581_mean_cov_4.28743545611**

```
CTCTCTTTTCTTCTTTAGTAGTCTTGCTAGCGGTCTATTAATTTTGTTGATCTTTTCAAAAAACCAGCTCCTGGATTCATTGACTTTTT
 >chr6:80059387-80059758 - E=1e-170
TGAAGGGTTTTTTGTGTGTCTCTCTCTTTCAGTTCTGCTCTGATCTGTTATTTCTTGCCTTCTGCTAGCTTTTTAATTTGTTTGCTCTT

GCTTCTCTAGTTTTTTTAATTGTGATATTACGGTGTCAATTTTAGATCTTTGCTGCTTTCTCTTGTGGGCACTTAGTGCTATAAATTTC

CCTCTACACACTGCTTTAAATGTGTCCCAGAGATTCTGGTATGTTAGTCTTTGTTCTCATTGGTTTCAAAGAACATCTTTATTTCTGCC

TTCATTTCATTAT|G|GATTATCTCCATGTTTCTAAAGACAGACCAAAACCAGAAGGTTACTTCCAAGATTTAATCTGTACCAACCAGT
              >chr6:91135864-91136076 - E=7e-116
CCAAATATTATTGGTTTTCAAGCATTCGATTTCCCTGCACAAAGAGCATGTACTTTCTTAACAGTGACTTGGCACTAAACTCCGTCTTC

AAATTTAGCTATGCAGTCTTGACTTTGAACTAGACAGGGGATTTCCATA
```
